# Identification of a presymptomatic and early disease signature for amyotrophic lateral sclerosis (ALS): protocol of the premodiALS study

**DOI:** 10.1186/s42466-025-00417-9

**Published:** 2025-08-19

**Authors:** Laura Tzeplaeff, Ana Galhoz, Clara Meijs, Lucas Caldi Gomes, Andrej Kovac, Amrei Menzel, Hatice Değirmenci, Abir Alaamel, Hüseyin Can Kaya, Ali Günalp Çelik, Sine Dinçer, Meltem Korucuk, Sibel Berker Karaüzüm, Elif Bayraktar, Vildan Çiftçi, Uğur Bilge, Filiz Koç, Antonia F. Demleitner, Anne Buchberger, Ricarda von Heynitz, Vincent Gmeiner, Christina Knellwolf, Mohammed Mouzouri, Joanne Wuu, A. Nazli Başak, Peter Munch Andersen, Florian Kohlmayer, Nicholas J. Ashton, Wojciech Kuban, Christof Lenz, Mary-Louise Rogers, Norbert Zilka, Philippe Corcia, Yossef Lerner, Markus Weber, Monika Turcanova Koprusakova, Hilmi Uysal, Michael Benatar, Michael P. Menden, Paul Lingor

**Affiliations:** 1 Department of Neurology, Rechts Der Isar Hospital of the Technical University Munich, Munich, Germany; 2 Department of Computational Health, Helmholtz Munich, Neuherberg, Germany; 3 Department of Biology, Ludwig-Maximilians University Munich, Munich, Germany; 4 Institute of Neuroimmunology, Slovak Academy of Sciences, Bratislava, Slovakia; 5 Bitcare GmbH, Technical University of Munich, Munich, Germany; 6 Department of Neurology, Akdeniz University Hospital, Antalya, Turkey; 7 Neuromuscular Center, Antalya Education and Research Hospital, Antalya, Turkey; 8 School of Medicine, Neurodegeneration Research Laboratory NDAL, Koç University, Research Center for Translational Medicine KUTTAM, Istanbul, Turkey; 9 Neurology Department, Çukurova University, Adana, Turkey; 10 Neuromuscular Diseases Center/ALS Clinic of the Kantonsspital St, Gallen, St. Gallen, Switzerland; 11 Centre de Reference SLA Et Autres Maladies du Neurone Moteur, Department of Neurology, CHRU Bretonneau, Tours, France; 12 Department of Neurology and ALS Center, University of Miami Miller School of Medicine, Miami, FL USA; 13 Department of Clinical Science, Neurosciences, Umeå University, Umeå, Sweden; 14 Department of Psychiatry and Neurochemistry, Sahlgrenska Academy at Gothenburg University, Gothenburg, Sweden; 15 Banner Alzheimer’s Institute and University of Arizona, Phoenix, AZ USA; 16 Banner Sun Health Research Institute, Sun City, Arizona, USA; 17 Department of Pharmacokinetics and Drug Metabolism, Maj Institute of Pharmacology of the Polish Academy of Sciences, Kraków, Poland; 18 Max Planck Institute for Biophysical Chemistry, Bioanalytical Mass Spectrometry, Göttingen, Germany; 19 Department of Clinical Chemistry, University Medical Center Göttingen, Göttingen, Germany; 20 Flinders Health and Medical Research Institute, College of Medicine and Public Health of the Flinders University, Adelaide, South Australia Australia; 21 Department of Neurology, Hadassah University Hospital-Ein Kerem, Jerusalem, Israel; 22 Department of Neurology, University Hospital Martin, Martin, Slovakia; 23 Jessenius Medical Faculty, Comenius University, Martin, Slovakia; 24 Department of Biochemistry and Pharmacology, Bio21 Molecular Science and Biotechnology Institute, The University of Melbourne, Parkville, VIC Australia; 25 German Center for Neurodegenerative Diseases (DZNE), Site Munich, Munich, Germany; 26 Munich Cluster for Systems Neurology (SyNergy), Munich, Germany

**Keywords:** Motoneuron disease, Pre-symptomatic, Multi-omic, Biomarkers, Early diagnosis, Observational study

## Abstract

**Introduction:**

The median time to diagnosis of amyotrophic lateral sclerosis (ALS) is approximately 12 months after the onset of first symptoms. This diagnostic delay is primarily due to the nonspecific nature of early symptoms and the clinical challenges in differentiating ALS from its mimics. Therefore, the discovery of reliable biomarkers for the early and accurate diagnosis of ALS represents a critical medical need.

**Methods:**

A total of 330 participants will be recruited across six international study sites. The cohort will include (1) pre-symptomatic gene mutation carriers, (2) symptomatic individuals up to 12 months after symptom onset with either ALS, ALS mimics, or a pure motor syndrome with yet unclear assignment, and (3) healthy controls. Participants will engage in a one-year longitudinal study, consisting of an initial evaluation at baseline visit and a follow-up visit 12 months later. Assessments will include an environmental and medical history questionnaire, neurological examinations, olfactory testing, cognitive/behavioral evaluations, and the collection of biological samples (serum, plasma, urine, tear fluid, and cerebrospinal fluid). Proteomic, metabolomic, and lipidomic analyses will be performed using mass spectrometry and targeted immunoassays, with all samples processed under standardized protocols. The resulting multimodal dataset will be systematically integrated in an effort to uncover a presymptomatic and early ALS signature.

**Perspective** The premodiALS study aim to identify a clinico-molecular signature characteristic of presymptomatic and early ALS. These findings may have relevance to early diagnosis and future clinical practice for ALS disease.

**Supplementary Information:**

The online version contains supplementary material available at 10.1186/s42466-025-00417-9.

## Introduction

Amyotrophic lateral sclerosis (ALS) is the most common adult-onset motor neuron disease. Despite advanced healthcare systems in industrialized countries, the average diagnostic delay for ALS remains approximately 12 months after symptom onset [1]. This delay is particularly critical given the median life expectancy of only 3 to 5 years post-symptom onset. Moreover, nearly half of ALS patients are initially misdiagnosed, largely due to limited familiarity with ALS among general practitioners and to the clinical challenges in distinguishing ALS from other disorders that mimic its early symptoms [1]. To improve and harmonize diagnosis, guidelines for motor neuron diseases have been published by the German Society of Neurology [2]. Given the rapid progression of the disease, the identification of reliable biomarkers for early and accurate diagnosis is essential to initiate timely therapy and facilitate patient enrollment in clinical trials.

Biomarker research in ALS has identified some promising candidates with potential for improving diagnosis and disease monitoring. Neurofilaments (Nf) are the most extensively studied biomarkers for ALS. Both phosphorylated Nf heavy chain (pNfH) and Nf light chain (NfL) levels in serum and cerebrospinal fluid (CSF) are increased in ALS compared to healthy controls and mimics, correlating with the rate of neuronal and axonal damage [3]. Recently, NfL was also shown to be a useful marker of therapy response [4]**.** Additionally, urinary molecules have been studied as markers of disease progression, including the soluble extracellular domain of the neurotrophin receptor p75 (sp75^ECD^) and neopterin [5]. Other proteins that have been identified to be differentially regulated in ALS cohorts include chitinases, chitinase-like proteins, creatine kinase, troponin, total Tau and p-Tau, collagen proteins, and several inflammatory markers such as MCP-1, IL-6, and IL-18 [5,6]. In addition to blood- or CSF-derived molecules, tear fluid may hold potential for biomarker discovery, as demonstrated in other neurodegenerative disorders [7].

Biomarkers have the potential to predict the timing of phenoconversion or identify sporadic patients in the presymptomatic phase of ALS, which refers to the period before the clinical manifestation of the disease. Although the majority of ALS cases are sporadic (sALS), approximately 10–15% can be attributed to a genetic cause (genetic ALS or gALS), even in the absence of a family history. However, almost half of gALS cases show a positive family history (fALS). In 50–70% of fALS cases, the cause of disease can be attributed to known mutations, such as repeat expansions in the *C9orf72* gene or mutations in the *SOD1*, *TARDBP* and *FUS* genes. Genetic testing of biological relatives of fALS cases can identify individuals who carry ALS-associated pathogenic variants but have not yet developed clinical manifestations of the disease, hereafter referred to as "pre-symptomatic gene mutation carriers" (PGMC).

Our hypothesis is that PGMC gradually accumulate molecular changes that eventually lead to the clinical manifestation of ALS. Previous studies demonstrated significant alterations in PGMC well before the manifestation of symptoms. For instance, neurofilaments are elevated in the serum and CSF of PGMC prior to phenoconversion [8]. Notably, elevated NfL levels have also been observed in non-mutation carriers who were later diagnosed with ALS, suggesting that NfL could serve as a susceptibility/risk marker in the early stages of the disease [9].

Alterations in cognition (e.g. verbal fluency), neuroimaging (e.g. volumetric changes in specific anatomical regions), electrophysiology (e.g. MUNIX), sleep (e.g. polysomnography), metabolic (e.g. resting metabolic rate) and proteomics have been observed in PGMC before symptom onset, highlighting early ALS pathophysiology [10–15]. Although group level differences may inform about disease mechanisms, they may not be suitable as susceptibility/risk or diagnostic markers. Therefore, biomarkers that operate at the level of an individual and predict susceptibility to developing clinically manifest disease are needed. The identification of disease biomarkers that precede axonal loss could help to explore their utility as diagnostic marker in patients in whom the ALS diagnosis is unclear. Such discovery could significantly transform ALS diagnosis and treatment. Pre-symptomatic clinical assessments and multi-omic biofluid profiling offer promising avenues for uncovering novel biomarker combinations, as seen in other neurodegenerative diseases like Alzheimer’s and Parkinson’s disease [16,17]. These approaches could enable the identification of individuals at risk when no genetic cause is identified, predict phenoconversion, provide an earlier diagnosis and finally improve access to clinical trials and treatments in ALS.

Here, we present the design of the premodiALS study, an international project focused on identifying a clinico-molecular signature that differentiates PGMC from healthy controls and ALS from ALS mimics, ultimately contributing to a deeper understanding of ALS pathophysiology.

## Methods

### Aim of the trial

**Fig. 1:**
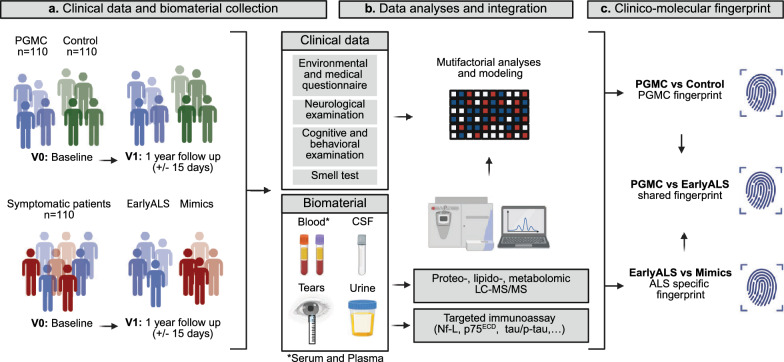
Overview of the premodiALS study procedures, analyses and outcomes. (**a**) Clinical and biomaterial collection from the PGMC, control and symptomatic patients’ groups. The symptomatic patients group includes subjects within 12 months of symptom onset with either early ALS or an ALS mimic, or a pure motor syndrome that does not permit to establish a certain diagnosis at the baseline visit (V0). The categorization into early ALS and ALS mimic groups will be determined a posteriori at the follow up visit (V1). (**b**) Analysis and integration of the clinical data and molecular data obtained from the biomaterial collection. (**c**) Identification of a clinico-molecular fingerprint of PGMC and early ALS. V: visit; PGMC: pre-symptomatic gene mutation carrier. *Created in BioRender. Tzeplaeff, L. (2025)*

The premodiALS study is a multicenter, prospective investigation aimed at identifying clinical markers and molecular biomarkers, and their combinations, that differentiate PGMC and early ALS cases from healthy controls and ALS mimics. Participants are assessed longitudinally at two time points: a baseline visit (V0) and a follow-up visit (V1) at an interval of one year (Fig. 1).

### Study description and study design

#### Participant description and eligibility criteria

Three groups of participants are included: (1) Pre-symptomatic gene mutation carriers (PGMC), (2) symptomatic subjects (SYMP) including either patients with a pure motor syndrome or early ALS or ALS mimics, and (3) controls (CTR).

1. PGMC are individuals who carry a pathogenic variant in a known ALS-associated gene but have not yet developed clinically manifest ALS. Genetic testing must have been conducted by a clinically certified laboratory.
2. The second group consists of symptomatic patients (SYMP) within 12 months after symptom onset. These may present either as early ALS (as per the El Escorial criteria) or as known ALS mimic or as patients with a pure motor syndrome, which is insufficient to establish an ALS diagnosis at the baseline visit (V0), and who may later develop into either ALS or an ALS mimic. ALS mimics refer to diseases that initially exhibit clinical symptoms resembling ALS, such as multifocal motor neuropathy, benign fasciculations, myopathies/myositides, radiculopathies, generalized myasthenia gravis, and others. The definite classification into “early ALS” or “ALS mimic” will be established at the follow-up visit (V1), when clinical progression provides diagnostic certainty.
3. Control (CTR) subjects can be family members of PGMC who are not carrying the genetic mutation, as well as life partners, spouses or friends, who are exposed to similar environmental factors, are within a comparable age range, and do not show any motor symptoms.

All groups include male and female subjects aged 18 years or older, provided that informed consent to participate in the study is obtained. Exclusion criteria include individuals who do not consent to biosampling or are unable to attend the V1 follow-up visit (12 months after baseline) at the study center. Subjects on anticoagulation therapy may participate, but they will be excluded from lumbar puncture.

#### Visit plan and assessments

Informed consent is obtained before any study-specific procedures are performed. A total of two visits (V0 and V1) at an interval of one year (± 15 days) are performed (Table 1). During the baseline V0, a questionnaire focused on demographics, environmental and medical history, as well as the ALS Functional Rating Scale—Revised (ALSFRS-R), is completed by the participant together with a trained study nurse or research coordinator to ensure that all questions are answered to avoid missing data (Additional file 1). For ALSFRS-R assessment, translated versions of the scale were used in each country, some of which have been validated previously. The baseline V0 assessment includes a detailed neurological examination; a cognitive and behavioral examination using the Edinburgh Cognitive and Behavioral ALS Screen (ECAS), including the caregiver/informant interview to detect behavioral abnormalities common in behavioral variant of FTD; as well as a standardized brief smell identification test (B-SIT). The questionnaire (Additional file 1) and the ECAS are provided in the participant's native language (i.e., German, Swiss-German, French, Slovak, Turkish, or Hebrew). Officially translated and culturally adapted version of the ECAS exist for all of these languages, except Turkish, which is currently adapted in the scope of the premodiALS study. The B-SIT is provided in either German, French, Turkish or English. To account for potential olfactory disturbances caused by SARS-CoV-2 infection, participants are asked about their history of SARS-CoV-2 infection and whether they have experienced any issues with their sense of smell. The collected biological samples include blood (serum, plasma), CSF, urine and tear fluid (collected with Schirmer test strips). Before CSF collection, coagulation parameters (e.g. the international normalized ratio, the partial thromboplastin time and platelet count) are analyzed. Participants with an altered coagulation state will be excluded from the lumbar puncture procedure, but will not be excluded from the study.

**Table 1:** Study visit schedule

Visit item	baseline V0	follow-up V1
Participant information and informed consent	X	
Questionnaire		
Part A: General aspects (e.g., handedness, home city size, family history of neurodegenerative diseases)	X	
Part B: Symptoms (e.g., motor and non-motor symptoms, ALS/FTD symptoms and non-ALS/FTD symptoms in the past 10 years)	X	
Part C: Contact with the health system (e.g., visit to specialists in neurology, orthopedics, cardiology, and others in the past 10 years)	X	
Part D: Pre-existing conditions (e.g., hypertension, depressive disorder, or other medical conditions in the past 10 years)	X	
Part E: Nutrition and weight (e.g., weight, diet, or fasting episodes in the past 10 years)	X	
Part F: Lifestyle, social aspects, special events (e.g., highest professional degree, caffeine and alcohol consumption, episodes of exceptional physical activity in the past 10 years)	X	
Part G: Special assessment of motor symptoms (ALSFRS-R)	X	X
Neurological examination	X	X
Cognitive and behavioral examination (ECAS)	X	X
Smell test (B-SIT)	X	X
Acquisition of blood samples		
Serum	X	X
EDTA Plasma	X	X
PAXGene RNA		X
EDTA whole blood collection (Only for *C9orf72* family background)		X
Acquisition of CSF samples	X	X
Acquisition of urine samples	X	X
Acquisition of tear fluid samples	X	X

During the follow-up V1, all assessments are repeated in the same way as for the baseline V0, with the exception of the questionnaire, where only the ALSFRS-R is repeatedly assessed.

In addition to the predefined sample collection, each participant may consent to donate additional biomaterial for biobanking during both baseline V0 and follow-up V1. This additional material is obtained simultaneously with the predefined biomaterial study collection and will be available for future collaborative projects beyond what is specified in the premodiALS protocol.

#### Biomaterial collection protocol

During each visit, biological samples, including serum, plasma, urine, tear fluid, and CSF are collected according to standard operating procedures. Detailed methods for biomaterial collection are provided in Additional file 2.

#### Recruitment

**Fig. 2:**
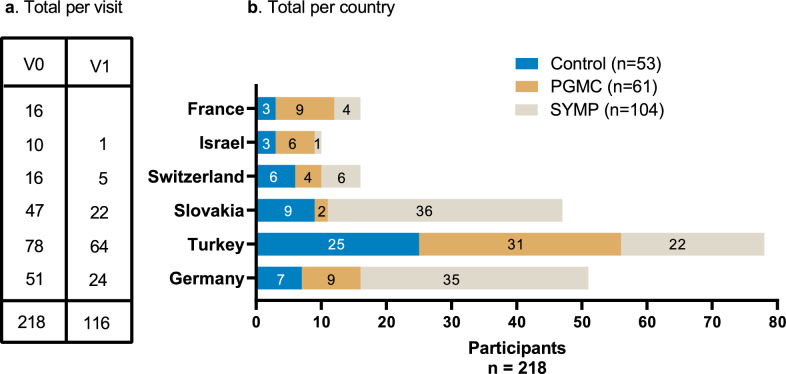
premodiALS recruitment status (as of 06/2025). (**a**) Number of participants recruited per country in the V0 and V1 for all groups. (**b**) Number of participants recruited per country in the control, PGMC and the symptomatic patient groups. V: visit; PGMC: pre-symptomatic gene mutation carrier; SYMP: symptomatic patients’ group (early ALS or ALS mimics or pure motor syndrome). Created in Graphpad Prism. *Tzeplaeff, L.*

Patients are recruited at 6 international study sites: Klinikum rechts der Isar of the Technical University of Munich, Munich, Germany; Akdeniz University Hospital, Antalya, Turkiye; Hadassah Ein Karem University Hospital, Jerusalem, Israel; Kantonsspital St.Gallen, St. Gallen, Switzerland; University Hospital Martin, Slovakia; and The CHRU of Tours, Tours, France. All recruitment centers are well-known ALS reference centers. The recruitment goal is a total of 110 PGMC, 110 control and 110 symptomatic patients. As of June 2025, recruitment is still ongoing in the 6 international study sites and a total of 218 participants have been recruited, including 58 CTR, 61 PGMC and 104 SYMP (Fig. 2 and Table 2).

**Table 2:** Current cohort description

	**Control**	**PGMC**	**SYMP**
**Participant number**	53	61	104
**Age (years)** Mean (± sd) Median (min–max)	47.1 (± 11.7) 48 (23–78)	48.3 (± 15.8) 47 (22–86)	59.3 (± 13.3) 61 (23–86)
**Sex**			
Female	24 (45.3%)	35 (57.4%)	35 (33.7%)
Male	29 (54.7%)	26 (42.6%)	69 (66.3%)
**Genetic mutations**			
*C9orf72*	-	27 (44.3%)	2 (1.9%)
*SOD1*	-	17 (27.9%)	4 (3.9%)
*TARDBP*	-	6 (9.8%)	1 (1.0%)
*FUS*	-	2 (3.3%)	-
Others	-	9 (14.7%)	2 (1.9%)

SYMP: symptomatic patients within 12 months after symptom onset with either ALS, ALS mimics or a pure motor syndrome that will be classified as ALS or mimic at the visit V1

### Outcome measures

#### Clinical data collection

Clinical data will be obtained through the neurological examination, the ECAS, the B-SIT, and the questionnaire. The neurological examination will include the following parameters: 1) cranial nerve status, tongue motility, dysarthria, dysphagia, 2) examination of the motor system with degrees of strength for neck flexion and extension, upper and lower extremities as well as muscle bulk and muscle tone, 3) testing of the deep tendon reflexes, the palmomental reflex, pathological reflexes, 4) examination of the sensory system, and 5) examination of the coordination, stance and gait. For symptomatic patients, we will attempt to classify the phenotype according to the OPM classification [18]. The B-SIT is composed of odors specific to the culture of the associated country language. The type and order of odors can therefore vary for different recruitment centers. For this reason, B-SIT data will be collected as a score of correct answers on a total of 12 different items. The premodiALS questionnaire is divided in 7 Parts (Parts A-G) and includes the following parameters: A) general aspects, B) symptoms, C) contact with the health system, D) pre-existing conditions, E) nutrition and weight, F) lifestyle, social aspects, special events, and G) assessment of motor symptoms (ALSFRS-R). The full questionnaire is available in Additional file 1.

#### Molecular analyses

The biomaterial from both visits will be subjected to proteomic, metabolomic and lipidomic profiling, as well as the examination of individual disease-relevant proteins (Table 2). These data will be generated in five analytical centers. Plasma/serum and CSF proteome analyses by high resolution mass spectrometry will be performed at the Maj Institute of Pharmacology, Warsaw, Poland. The tear fluid proteome will be analyzed by high resolution mass spectrometry at the University Medical Center Göttingen, Göttingen, Germany. Metabolomic and lipidomic data will be obtained from plasma, urine and CSF via liquid chromatography tandem mass spectrometry (LC–MS/MS) at the Institute of Neuroimmunology, Slovak Academy of Sciences, Bratislava, Slovakia. Targeted immunoassays will be used for the examination of individual disease-relevant proteins. In urine samples, sp75^ECD^ and neopterin will be analyzed with an enzyme-linked immunosorbent assay (ELISA) at the Flinders University, Adelaide, South Australia. Plasma/serum and CSF samples will be subjected to measurements of NfL, GFAP, total-Tau/phospho-Tau, as well as a panel of synaptic, lysosomal and inflammatory proteins using the digital ELISA based on single molecule arrays (SIMOA) and the Nucleic acid Linked Immuno-Sandwich Assay (NULISA) at the Sahlgrenska Academy at Gothenburg University, Mölndal Sweden (Table 3).

**Table 3:** Summary of expected molecular outcome measures

Source sample type	Proteomics	Metabolomics/ lipidomics	Targeted biomarkers
Blood			
Plasma	X	X	X
Serum	X		X
Urine		X	X
CSF	X	X	X
Tear fluid	X		

Additional biomaterial (serum, plasma, CSF and urine) stored in the biobank and not yet allocated to predefined analyses will be available for collaborative projects.

#### Statistical analyses

To identify a clinico-molecular fingerprint, we will initially analyze each dataset separately. In a subsequent step, we will integrate the relevant features to increase statistical power to identify robust signatures. Longitudinal difference between the baseline V0 and the follow-up V1 visit will be assessed for each group. Furthermore, we will systematically compare PGMC vs. CTR and early ALS vs. ALS mimics, adjusting for sex and age. The fingerprint of PGMC will also be compared with early ALS data to determine changes common to both groups. To describe the potentially strongest contrast, we will also compare early ALS and CTR groups. Where applicable, data will be stratified by sex and/or genetic mutation, or adjusted for these variables.

Clinical data will be comprehensively analyzed using both univariate and multivariate statistical methods to identify disease-specific clinical features. In the univariate framework, continuous and categorical variables from the questionnaire will be tested separately. Analyses will first be performed on the total data set with sex and age adjustment. In parallel, data will be stratified by sex and results will be reported using sex-adjusted odds ratio. Significance will be evaluated using Fisher’s exact test for categorical variables and the Wald test for continuous variables, respectively. Additionally, multivariate generalized linear models will be employed to predict PGMC and early ALS participants, with sequential extraction of predictive features. Statistical findings will be corrected for multiple hypothesis testing utilizing the Benjamini–Hochberg correction at 5% False Discovery Rate (FDR).

Molecular data will be analyzed to identify differentially enriched proteins and metabolites. In concordance with clinical data analysis, the same 5% FDR cut-off will be employed. Gene Set Enrichment Analysis will be used for enrichment analysis with Gene Ontology annotations via the clusterProfiler package (v4.6.2). Given the heterogeneous nature of ALS, we will apply data-driven clustering methods and assess robustness with Akaike’s Information Criterion, Silhouette coefficient, and Dunn index. Results from the metabolomic and proteomic analysis will be compared with results from the MAXOMOD consortium [19]. Additionally, we will use the Wppi Bioconductor package to identify novel ALS-relevant proteins by constructing a protein–protein interaction using Omnipath, which will be explored via a random walk with restart algorithm to identify new biomarker candidates.

We will apply Gaussian mixture models, spectral bi-clustering and Multi‐Omics Factor Analysis to integrate clinical and molecular data to derive a robust presymptomatic and early ALS clinical-molecular fingerprint. Where applicable, the results will be corrected for the ALSFRS-R slope to take into consideration the different progression rates of the disease. Key features of this fingerprint will be replicated in an independent PGMC cohort from the *Pre-fALS* study (University of Miami, Miami, FL, USA) [20]. In addition, supervised machine learning models (i.e., linear regressions, random forest, and deep learning models) will be employed to predict disease progression, mitigating the risk of overlooking critical biomarkers. These models will be cross-validated, and predictive features will be systematically identified and assessed.

### Contact

Investigator initiated trial; P. Lingor (Principal Investigator); paul.lingor@tum.de.

## Perspective

With this study we aim to describe a clinico-molecular fingerprint and to provide supervised machine learning models capable of differentiating presymptomatic subjects and patients with ALS from ALS mimics and controls. This could facilitate early diagnosis and treatment and provide further insights into the disease mechanisms of this devastating neurodegenerative condition. One of the limitations of the study is the short follow-up period of one year. However, given the successful recruitment so far, we aim to extend the follow-up for another three years, including additional visits V2, V3 and V4 at yearly intervals. The additional follow-up visits will be conducted in accordance with the procedures detailed for visit V1 in this protocol. We assume that ALS develops through gradual molecular changes and not according to the punctuated equilibrium principle (i.e., motor dysfunction develops suddenly and without antecedent). However, if PGMC are many years from phenoconversion, these changes may not yet be apparent. In this case we will focus on emerging ALS cases within the PGMC cohort (phenoconverters). Comparing the baseline visit (V0) and follow-up visit (V1) of phenoconverters will identify key features of ALS phenoconversion.

Although premodiALS is a non-interventional, prospective observational study, subjects participating in the study may benefit from data obtained during the visits. As an example, the results of the olfactory testing can be communicated directly to the participants, so that a further evaluation of a possibly existing olfactory disorder can take place if desired. Furthermore, participants will receive a detailed neurological examination, allowing pathological changes to be detected and thus treated earlier, even if originating from other causes. The results of the biomaterials data will not be communicated to the participants, as they are only carried out at a later date and are usually only analyzed and meaningful as a group comparison. However, the study may identify a signature that could be used in the future to prioritize PGMC for a clinical trial of disease-modifying therapies.

Finally, while some biomaterial will exclusively be allocated for analyses predetermined in the premodiALS project, others will be biobanked and available for collaborative projects and future analyses. The premodiALS project presented here will provide a copious data source that could be compared to similar initiatives, such as *Pre-fALS*, GENFI or the EPIC studies [15,20,21]. With the premodiALS protocol, we therefore also aim to promote collaboration, encourage harmonization of protocols across studies to enable comparison of data across larger cohorts, thereby advancing scientific understanding and facilitating more robust research outcomes in the field.

## Supplementary Information

Additional file 1.

Additional file 2.

## Data Availability

Not applicable. However, to disseminate the design and results of the study, a website has been created, which is updated by regular news sections [22]. Scientific results will be disseminated in peer-reviewed, international journals, and at national and international conferences respecting the privacy of the participants.

## Abbreviations

ALS: Amyotrophic lateral sclerosis
Nf: Neurofilament
pNfH: phosphorylated Neurofilament, heavy chain
NfL: Neurofilament, light chain
CSF: Cerebrospinal fluid
sp75^ECD^: Soluble extracellular domain of the neurotrophin receptor p75
sALS: sporadic ALS
gALS: genetic ALS
fALS: familial ALS
PGMC: Pre-symptomatic gene mutation carrier
V0: Baseline visit
V1: Follow-up visit
CTR: Control
SYMP: Symptomatic
ALSFRS-R: ALS Functional Rating Scale - Revised
ECAS: Edinburgh Cognitive and Behavioral ALS Screen
B-SIT: Brief smell identification test
LC-MS/MS: Liquid chromatography tandem mass spectrometry
ELISA: Enzyme-linked immunosorbent assay
SIMOA: Single molecule array
NULISA: Nucleic acid Linked Immuno-Sandwich Assay
FDR: False Discovery Rate
