# Supplementary material for: Identification of a presymptomatic and early disease signature for amyotrophic lateral sclerosis (ALS): protocol of the premodiALS study
Source: Neurol Res Pract. 2025 Aug 19;7(1):56. doi: 10.1186/s42466-025-00417-9 (PMC12366133; doi:10.1186/s42466-025-00417-9)
Supplement: Supplementary file 2 — Additional file 2. [file 42466_2025_417_MOESM2_ESM.docx]

**Additional file 2**

### *Biomaterial collection protocol*

All biomaterial samples are collected on ice, then processed and frozen at -80°C as quickly as possible, within 30 to 60 minutes after collection.

Blood collection: During each visit, venous blood is drawn in 2 x 10 mL clot activator tube serum vacutainers (BD, 367896) for serum collection and in 2 x 10 mL EDTA K2 vacutainers (BD, 367525) for plasma collection. After collection, tubes are gently inverted 10 times, centrifuged at ≤1300 g for 10 minutes at 18–25°C. Supernatants are then aliquoted into 12 tubes of 500 µL each, and stored at -80°C until further use of the biomaterial. In addition, 3 mL of blood is collected into a citrate tube and 2.6 mL of blood is collected in an EDTA monovette tube. These two tubes of blood are shipped to the local laboratory to determine coagulation parameters before lumbar puncture for CSF collection. During the follow up visit (V1), 1 x 2.5 mL of blood is collected in a PAXgene RNA tube (BD, 762165) for isolation and purification of intracellular RNA. Tubes are inverted 10 times, kept in an upright position at room temperature for 2 hours, transferred first to a -20°C freezer for approximately 24 hours as required by the manufacturer, and finally transferred to a -80°C freezer, and kept at this temperature until shipment. If participants belong to a family with a *C9orf72* expansion genetic background, two tubes of 7.5 mL EDTA for whole blood will be additionally collected during the follow up visit (V1) and stored at -80°C.

CSF collection: After verification that the participant is not taking any anticoagulants and an unremarkable coagulation test the lumbar puncture is performed and 15 mL of CSF are collected in 2 polypropylene tubes. One tube is centrifuged at 2000 g for 10 minutes at 4°C, then supernatant is aliquoted into 14 tubes of 500 μL and kept at -80°C until further usage. The second tube is sent to the local laboratory for basic CSF examination to quantify white cell counts, lactate, total protein and to exclude blood contamination.

Urine collection: During each visit, participants are asked to dispense approximately 50 mL of urine into a urinalysis container. A urinalysis strip (UriScan™) is used to assess blood, bilirubin, urobilinogen, ketone, protein, nitrite, glucose, specific gravity, leukocytes and pH. From the urinalysis container, 15 mL of urine is collected in a 15 mL tube and centrifuged at 2000 g for 10 min at 4°C. Supernatant is then distributed into 8 aliquots of 500 μL and stored at -80°C until further usage.

Tear fluid collection: Schirmer test strips (MDT, TearFlo™) are inserted at the medial and lateral thirds of the lower eyelid of each eye. Collection lasts 5 minutes with eyes closed ^[1]^. As the tear volume highly varies between individuals, the wetting length of each stripe is documented. Indeed, different wetting lengths can result in different protein concentrations and therefore impact analyses if not normalized. Stripes are individually placed in 500 μL tubes and stored at -80°C until usage.

1. Luib, E., Demleitner, A. F., Cordts, I., Westenberg, E., Rau, P., Pürner, D., Haller, B., & Lingor, P. (2024). Reduced tear fluid production in neurological diseases: A cohort study in 708 patients. *Journal of Neurology*, *271*(4), 1824–1836. https://doi.org/10.1007/s00415-023-12104-3
